# Age-related alterations of brain metabolic network based on [18F]FDG-PET of rats

**DOI:** 10.18632/aging.203851

**Published:** 2022-01-25

**Authors:** Xin Xue, Jia-Jia Wu, Bei-Bei Huo, Xiang-Xin Xing, Jie Ma, Yu-Lin Li, Mou-Xiong Zheng, Xu-Yun Hua, Jian-Guang Xu

**Affiliations:** 1 School of Rehabilitation Science, Shanghai University of Traditional Chinese Medicine, Shanghai 201203, China; 2 Department of Rehabilitation Medicine, Yueyang Hospital of Integrated Traditional Chinese and Western Medicine, Shanghai University of Traditional Chinese Medicine, Shanghai 200437, China; 3 Department of Traumatology and Orthopedics, Yueyang Hospital of Integrated Traditional Chinese and Western Medicine, Shanghai University of Traditional Chinese Medicine, Shanghai 200437, China; 4 Engineering Research Center of Traditional Chinese Medicine Intelligent Rehabilitation, Ministry of Education, Shanghai 201203, China

**Keywords:** aging, PET, brain metabolic network, topological property, network robustness

## Abstract

Using animal models to study the underlying mechanisms of aging will create a critical foundation from which to develop new interventions for aging-related brain disorders. Aging-related reorganization of the brain network has been described for the human brain based on functional, metabolic and structural connectivity. However, alterations in the brain metabolic network of aging rats remain unknown. Here, we submitted young and aged rats to [18F]fluorodeoxyglucose with positron emission tomography (18F-FDG PET) and constructed brain metabolic networks. The topological properties were detected, and the network robustness against random failures and targeted attacks was analyzed for age-group comparison. Compared with young rats, aged rats showed reduced betweenness centrality (*BC*) in the superior colliculus and a decreased degree (*D*) in the parietal association cortex. With regard to network robustness, the brain metabolic networks of aged rats were more vulnerable to simulated damage, which showed significantly lower local efficiency and clustering coefficients than those of the young rats against targeted attacks and random failures. The findings support the idea that aged rats have similar aging-related changes in the brain metabolic network to the human brain and can therefore be used as a model for aging studies to provide targets for potential therapies that promote healthy aging.

## INTRODUCTION

As we age, our brains undergo natural structural, chemical and functional deterioration, along with strong cognitive decline, characterized by brain atrophy, blood flow reductions, synaptic degeneration and neurochemical alternations [1]. Such human studies, however, rarely exclude common health conditions of aging, such as hypertension, that could influence the accuracy of the results [2]. Therefore, it is necessary to further understand age-related brain changes in nonhuman animal models to distinguish normal aging from pathological brain abnormalities. Using animal models to study the underlying mechanisms of aging will therefore provide a critical foundation from which to develop new interventions for aging-related brain disorders.

Normal aging has been associated with considerable alterations to the brain network and its associated functions [3–5]. Aging-related reorganization of the brain network has been described for the human brain based on functional, metabolic and structural connectivity. Age-related declines in functional connectivity have been observed both within and between several resting-state networks, such as the default mode, salience, dorsal attention and sensorimotor networks [4]. Moreover, brain networks in the elderly show decreased modularity and efficiency and exhibit a degeneration process in which the aging brain system shifts from a small-worldness network to a regular network along with normal aging [6, 7]. In addition, age-related alterations in structural network metrics are similar to the findings of functional connectivity studies [1].

[18F]Fluorodeoxyglucose with positron emission tomography (18F-FDG PET) is a valuable tool for measuring energy consumption in neurons, which reflects neuronal communication signals [8]. Thus, 18F-FDG PET is considered a functional neuroimaging technique for detecting age-related brain activity changes. Over the past few decades, many studies have used 18F-FDG PET to investigate the changes in brain mechanisms associated with normal aging. Overall, a consistent finding in most studies is a significant age-related decline in glucose metabolism observed in the frontal lobe [9, 10]. Decreased glucose uptake may reflect tissue loss or shrinkage [3]. In addition, age-related changes also appear in metabolic brain networks, *e.g.,* increased clustering, decreased efficiency, reduced robustness and changed nodal centralities in association and paralimbic cortex regions [7].

However, it has not been definitively reported whether the age-related changes in glucose metabolism are consistent between the rat brain and the human brain, especially in terms of metabolic brain networks. It remains unclear whether the brain metabolic network of aged rats has similar age-related changes to the human brain.

In the present study, we submitted young and aged rats to 18F-FDG PET and constructed brain metabolic networks. The topological properties were detected, and the network robustness against random failures and targeted attacks was analyzed for age-group comparisons.

## RESULTS

### Differences in metabolic connectivity of the brain metabolic networks of aged and young rats

**Figure 1:**
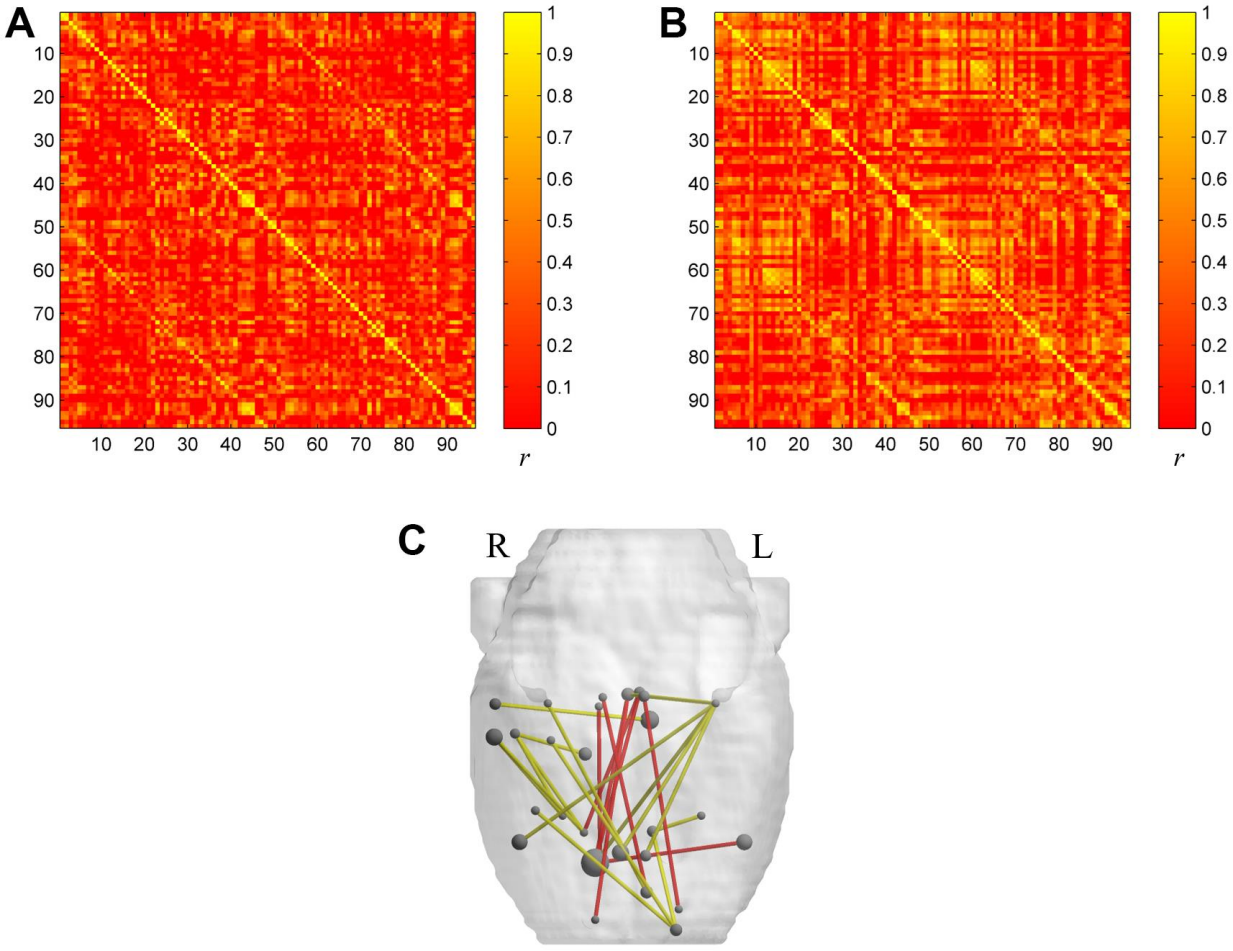
The metabolic brain networks of the two groups (**A** for the aged group and **B** for the young group). The color bar indicates the Pearson correlation coefficient between each pair of brain regions. The rank and row successively represent the 96 brain regions (Table 4). (**C**) The 3D Figure represents metabolic connections with significant differences between the two groups. Metabolic connections are overlaid on an anatomical map using nodes and edges. The red line shows significantly increased metabolic connectivity in the aged group (p<0.001) compared with the young group. The yellow line shows significantly reduced metabolic connectivity in the aged group (p<0.001) compared with the young group.

All of the rat brain regions included in the study are listed in Table 1. The group-level metabolic correlation matrix is shown in Figure 1 (A, the aged group; B, the young group). Compared to young rats, significantly decreased metabolic connectivity between regions related to visual (left visual cortex, right visual cortex), auditory (right auditory cortex), and olfactory (right entorhinal cortex) senses, as well as significantly increased metabolic connectivity between limbic brain regions (right raphe, left raphe, right nucleus accumbens core, right nucleus accumbens shell), were observed in aged rats (Table 2 and Figure 1). All significance tests were conducted at the threshold p < 0.001 without correction.

**Table 1:** All of the rat brain regions included in the study.

**No.**	**Brain regions**	**Abbreviation**	**No.**	**Brain regions**	**Abbreviation**
1	Nucleus Accumbens Core_R	AcbC_R	49	Nucleus Accumbens Core_L	AcbC_L
2	Nucleus Accumbens Shell_R	AcbSh_R	50	Nucleus Accumbens Shell_L	AcbSh_L
3	Amygdala_R	Amy_R	51	Amygdala_L	Amy_L
4	Bed Nucleus of the Stria Terminalis_R	BNST_R	52	Bed Nucleus of the Stria Terminalis_L	BNST_L
5	Caudate Putamen_R	CPu_R	53	Caudate Putamen_L	CPu_L
6	Corpus Collosum_R	CoC_R	54	Corpus Collosum_L	CoC_L
7	Cortex- Auditory_R	Aud_R	55	Cortex- Auditory_L	Aud_L
8	Cortex- Cingulate_R	CiC_R	56	Cortex- Cingulate_L	CiC_L
9	Cortex- Entorhinal_R	EC_R	57	Cortex- Entorhinal_L	EC_L
10	Cortex- Frontal Association_R	FrA_R	58	Cortex- Frontal Association_L	FrA_L
11	Cortex- Insular_R	In_R	59	Cortex- Insular_L	In_L
12	Cortex-Medial Prefrontal_R	mPFC_R	60	Cortex-Medial Prefrontal_L	mPFC_L
13	Cortex- Motor_R	M1_R	61	Cortex- Motor_L	M1_L
14	Cortex- Orbitofrontal_R	OFC_R	62	Cortex- Orbitofrontal_L	OFC_L
15	Cortex- Parietal Association_R	ParA_R	63	Cortex- Parietal Association_L	ParA_L
16	Piriform Cortex_R	PC_R	64	Piriform Cortex_L	PC_L
17	Cortex- Retrosplenial_R	RSC_R	65	Cortex- Retrosplenial_L	RSC_L
18	Cortex- Somatosensory_R	S1_R	66	Cortex- Somatosensory_L	S1_L
19	Cortex- Temporal Association_R	TeA_R	67	Cortex- Temporal Association_L	TeA_L
20	Cortex- Visual_R	V1_R	68	Cortex- Visual_L	V1_L
21	Diagonal Band_R	DB_R	69	Diagonal Band_L	DB_L
22	Globus Pallidus_R	GPa_R	70	Globus Pallidus_L	GPa_L
23	Antero-Dorsal Hippocampus_R	adHIP_R	71	Antero-Dorsal Hippocampus_L	adHIP_L
24	Posterior Hippocampus_R	pHIP_R	72	Posterior Hippocampus_L	pHIP_L
25	Postero-Dorsal Hippocampus_R	pdHIP_R	73	Postero-Dorsal Hippocampus_L	pdHIP_L
26	Hippocampus Subiculum_R	sHIP_R	74	Hippocampus Subiculum_L	sHIP_L
27	Ventral Hippocampus_R	vHPC_R	75	Ventral Hippocampus_L	vHPC_L
28	Lateral Hypothalamus_R	LH_R	76	Lateral Hypothalamus_L	LH_L
29	Medial Hypothalamus_R	MH_R	77	Medial Hypothalamus_L	MH_L
30	Internal Capsule_R	Ic_R	78	Internal Capsule_L	Ic_L
31	Interstitial Nucleus of the Posterior Limb of the Anterior Commissure_R	IPAC_R	79	Interstitial Nucleus of the Posterior Limb of the Anterior Commissure_L	IPAC_L
32	Medial Geniculate_R	MG_R	80	Medial Geniculate_L	MG_L
33	Mesencephalic Region_R	MR_R	81	Mesencephalic Region_L	MR_L
34	Olfactory Nuclei_R	ON_R	82	Olfactory Nuclei_L	ON_L
35	Olfactory Tubercle_R	OT_R	83	Olfactory Tubercle_L	OT_L
36	Periaqueductal Grey_R	PAG_R	84	Periaqueductal Grey_L	PAG_L
37	Pons_R	Pons_R	85	Pons_L	Pons_L
38	Raphe_R	Raphe_R	86	Raphe_L	Raphe_L
39	Septum_R	Septum_R	87	Septum_L	Septum_L
40	Substantia Innominata_R	SI_R	88	Substantia Innominata_L	SI_L
41	Substantia Nigra_R	SN_R	89	Substantia Nigra_L	SN_L
42	Superior Colliculus_R	SC_R	90	Superior Colliculus_L	SC_L
43	Dorsolateral Thalamus_R	DLT_R	91	Dorsolateral Thalamus_L	DLT_L
44	Dorsal Midline Thalamus_R	dMT_R	92	Dorsal Midline Thalamus_L	dMT_L
45	Ventromedial Thalamus_R	VMT_R	93	Ventromedial Thalamus_L	VMT_L
46	Ventral Pallidum_R	VP_R	94	Ventral Pallidum_L	VP_L
47	Ventral Tegmental Area_R	VTA_R	95	Ventral Tegmental Area_L	VTA_L
48	Zona Incerta_R	ZI_R	96	Zona Incerta_L	ZI_L

**Table 2:** Significant differences in metabolic connectivity between regions.

**Aged group < young group**				**Aged group > young group**		
**Brain region**	**Brain region**	***P* values**		**Brain region**	**Brain region**	***P* values**
V1_L	AcbC_R	<0.001		Raphe_R	AcbC_R	<0.001
V1_L	CiC_R	<0.001		Raphe_R	ON_R	<0.001
V1_L	CiC_L	<0.001		Raphe_L	AcbC_R	<0.001
V1_L	Raphe_R	<0.001		Raphe_L	VP_R	<0.001
V1_L	In_R	<0.001		AcbSh_R	MR_R	<0.001
V1_R	mPFC_L	<0.001		AcbSh_R	In_L	<0.001
Aud_R	IPAC_R	<0.001		SC_R	mPFC_L	<0.001
Aud_R	VP_R	<0.001		OFC_L	PAG_L	<0.001
EC_R	IPAC_R	<0.001				
EC_R	VP_R	<0.001				
EC_R	ZI_R	<0.001				
FrA_L	S1_R	<0.001				
FrA_L	ParA_R	<0.001				
FrA_L	DB_L	<0.001				
TeA_R	VTA_L	<0.001				
DB_L	IPAC_L	<0.001				

V1, Cortex- Visual; AcbC, Nucleus Accumbens Core; CiC, Cortex- Cingulate; In, Cortex- Insular; mPFC, Cortex-Medial Prefrontal; IPAC, Posterior Limb of the Anterior Commissure; VP, Ventral Pallidum; ZI, Zona Incerta; FrA, Cortex- Frontal Association; S1, Cortex- Somatosensory; ParA, Cortex- Parietal Association; DB, Diagonal Band; TeA, Cortex- Temporal Association; VTA, Ventral Tegmental Area; AcbSh, Nucleus Accumbens Shell; SC, Superior Colliculus; OFC, Cortex- Orbitofrontal; ON, Olfactory Nuclei; MR, Mesencephalic Region; PAG, Periaqueductal Grey.

### No intergroup differences in global topological properties

**Figure 2:**
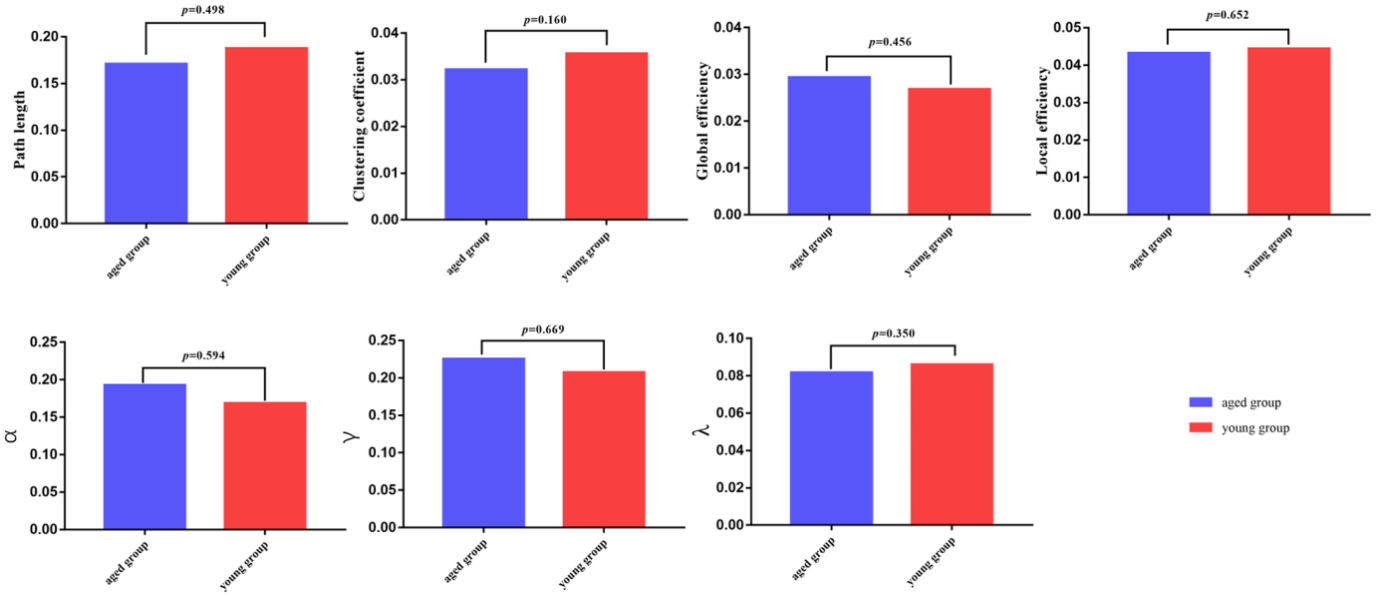
**Global parameters are displayed in the bar chart, with blue bars for the aged rats and red bars for the young rats.** In all of the parameters, no significant differences were found between the aged group and young group.

**Figure 3:**
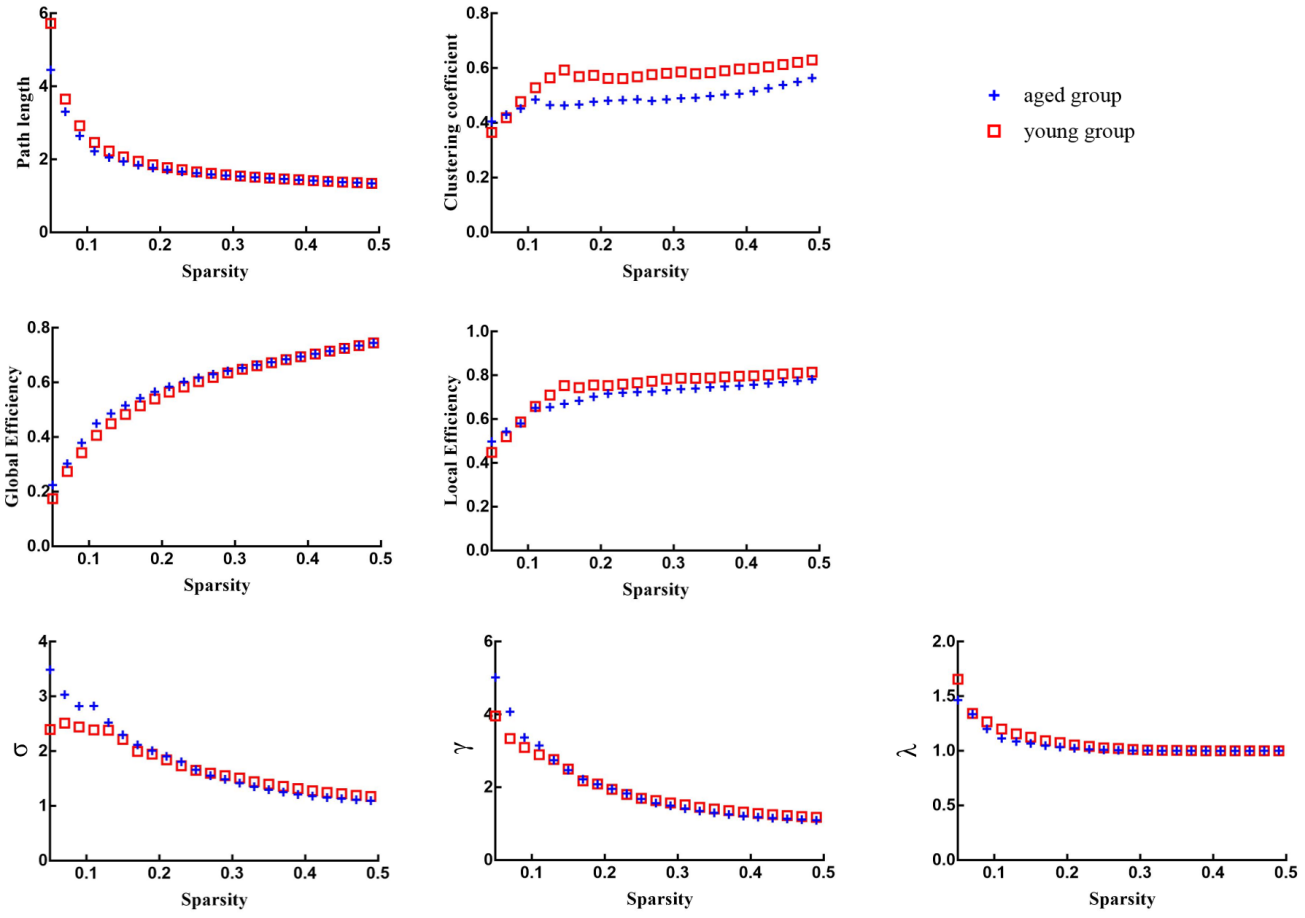
Global network properties of aged rats and young rats across a specific range of sparsity (0.05-0.5) at an interval of 0.01.

There were no significant differences in global network properties (path length *(Lp)*, clustering coefficient *(Cp)*, global efficiency *(E_glob_)*, or local efficiency *(E_loc_)*, *σ*, *γ*, *λ*) between aged rats and young rats (*p* > 0.05) (Table 3 and Figures 2, 3).

**Table 3:** Intergroup differences of global network properties.

**Global network properties**	***p* values**
Path length	0.498
Clustering coefficient	0.160
Global efficiency	0.456
Local efficiency	0.652
σ	0.594
γ	0.669
λ	0.350

### Lower regional topological properties of the aged brain metabolic network

**Figure 4:**
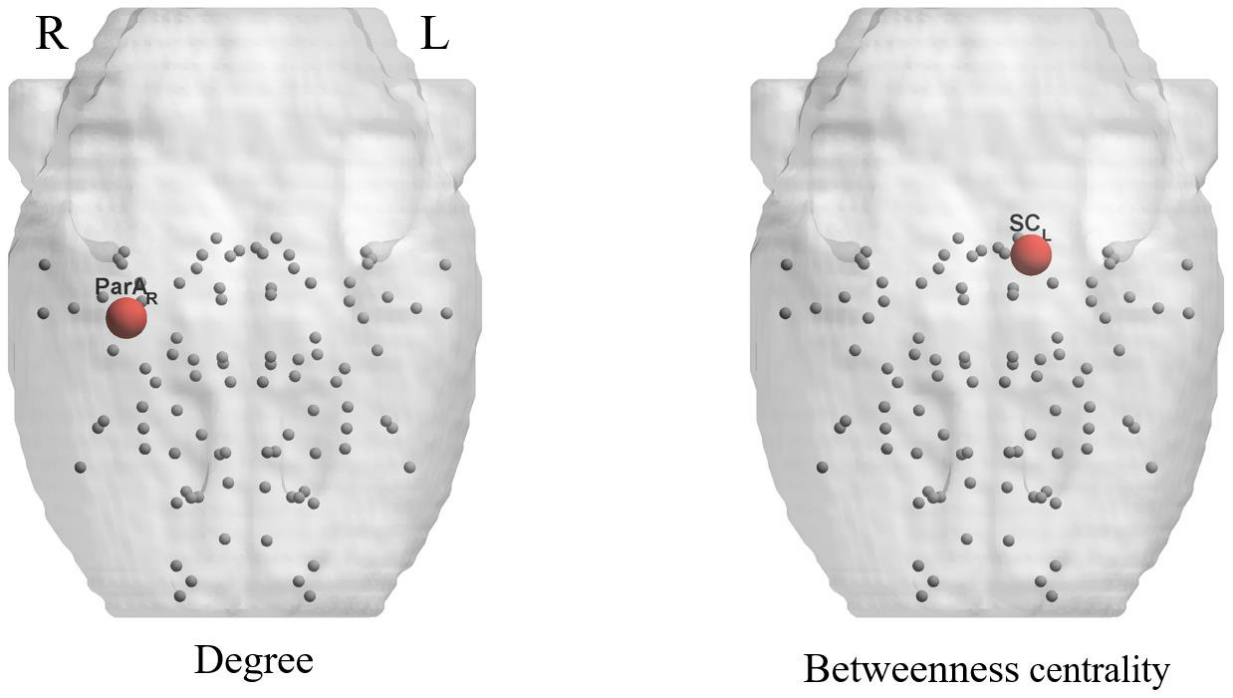
**Significant differences in nodal parameters are shown by 3D schematic Figures, corresponding to Table 3.** Red nodes indicate significant decreases in the aged group compared with the young group.

Intergroup differences of the regional network parameters are listed in Table 4. Compared with young rats, aged rats showed significantly lower betweenness centrality *(BC)* in the left superior colliculus (*p* < 0.001) and a lower degree *(D)* in the right parietal association cortex (*p* < 0.001) (Figure 4).

**Table 4:** Brain regions show significant differences in any of the three nodal characteristics.

**Brain regions**	***p* values**		
	**Betweenness centrality**	**Degree**	**Efficiency**
**Young rats>Aged rats**			
Cortex_Parietal_Association_R	-	<0.001	-
Superior_Colliculus_L	<0.001	-	-

### Lower robustness to virtual attacks in the aged brain metabolic network

Compared with aged rats, the networks of young rats were generally more robust to random failure and targeted attack in order of nodal *BC*.

### Random failure analysis

**Figure 5:**
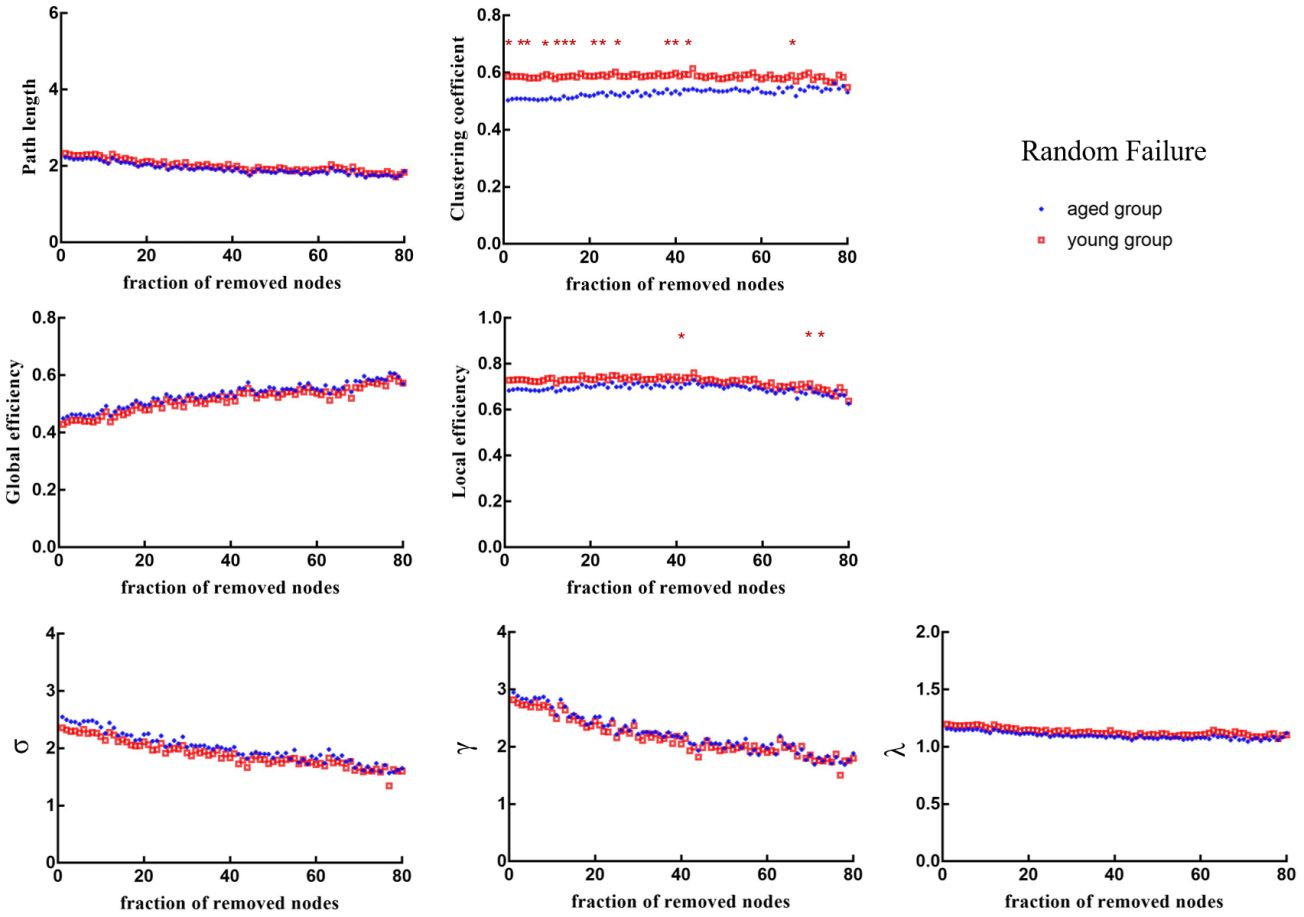
**Changes in topological properties of the global network of the remaining network after random failure.** The red * marker represents significant differences between aged rats and young rats.

When 1%, 4%, 5%, 9%, 13%, 15%, 17%, 22%, 24%, 27%, 39%, 41%, 44% and 67% of nodes were randomly removed, the *Cp* of brain networks in aged rats was significantly lower than that in young rats (*p* < 0.05). When 41%, 70% and 73% of nodes were randomly removed, the *E_loc_* of brain networks in aged rats was significantly lower than that in young rats (*p* < 0.05) (Figure 5).

### Targeted attack analysis

**Figure 6:**
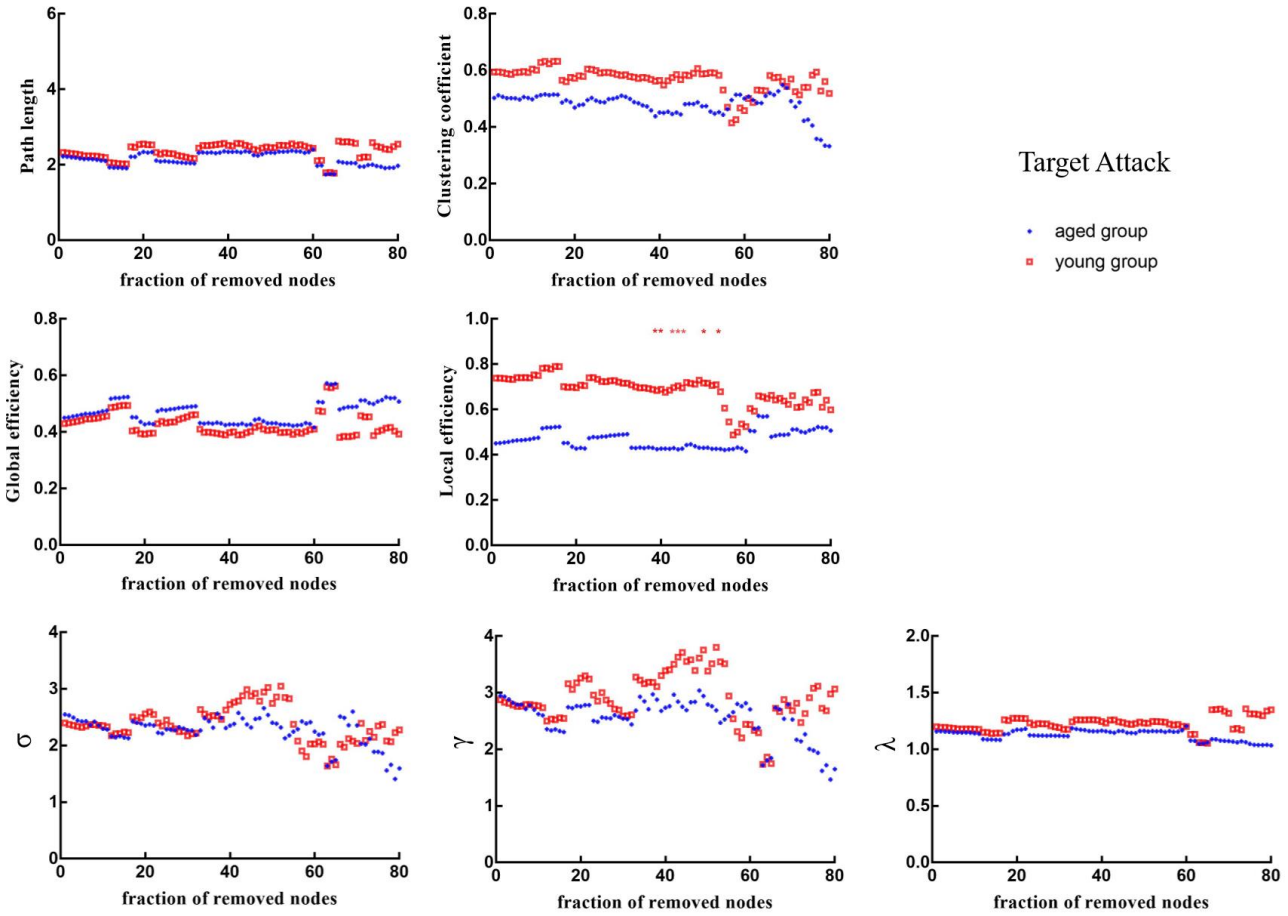
**Changes in topological properties of the global network of the remaining network after target attack in order of nodal betweenness centrality.** The red * marker represents significant differences between aged rats and young rats.

**Figure 7:**
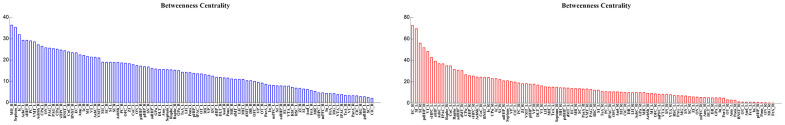
Hubs in decreasing order of betweenness centrality in the aged group (blue) and the young group (red).

**Figure 8:**
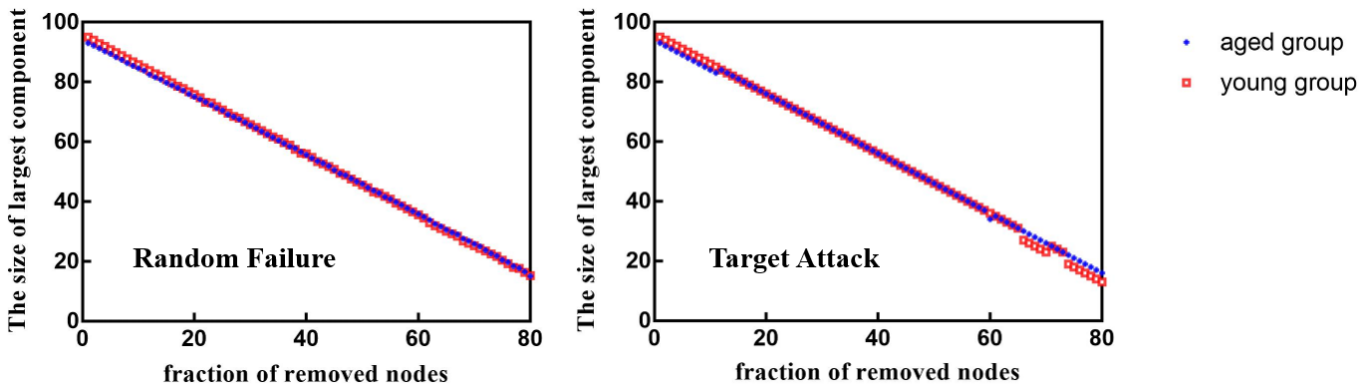
Between-group differences in network resilience to target attack and random failure.

When 39%, 40%, 43%, 44%, 45%, 50% and 53% of nodes were removed in order of nodal *BC*, the *E_loc_* of brain networks in aged rats was significantly lower than that in young rats (*p* < 0.05) (Figure 6). Figure 7 shows the brain regions in descending order of *BC*. We also calculated the size of the largest remaining component when nodes were continuously removed. When the nodes were removed, the size of the largest connecting component in both groups steadily and approximately decreased (*p* > 0.05) (Figure 8).

## DISCUSSION

In the current study, we investigated differences in the brain metabolic network between aged rats and young rats based on 18F-FDG PET imaging. Our main results were as follows: (1) Compared to young rats, metabolic connectivity between regions related to visual, auditory, and olfactory senses was significantly reduced in aged rats, as well as significantly increased between limbic brain regions; (2) For global network properties, there were no significant differences between aged rats and young rats (*p* > 0.05); (3) In terms of regional nodal characteristics, the aged rats showed significantly lower *BC* in the left superior colliculus (*p* < 0.001) and lower degree in the right parietal association cortex (*p* < 0.001); (4) With regard to network robustness, the brain metabolic networks of aged rats were more vulnerable to simulated damage, which showed significantly lower *E_loc_* and *Cp* than those of young rats against targeted attacks and random failures.

Degenerative changes caused by aging may affect the sensory experience, including vision, auditory and related cognitive domains [11]. Such changes occur as a part of normal physiological processes and may be irreversible [12]. In this study, the results demonstrated a decrease in metabolic connectivity between brain regions related to sensory experience that provided a basis to explain the pathogenesis of age-associated decline in sensory experience. However, the metabolic connectivity between emotion-related brain regions, such as raphe nuclei and nucleus accumbens, was significantly enhanced in aged rats. Raphe nuclei degeneration is related to the neurobiology of depression and is a common disorder in old age [13, 14]. Abnormal activation of raphe nuclei in metabolic connectivity might provide a new perspective for exploring the mechanism of depression in late life. The nucleus accumbens core, a limbic and premotor system nexus region, directly regulates behavior related to reward and motivation [15]. Consistent with the findings of this study, despite cognitive deficits, older adults have been reported to be sensitive to affective manipulations, such as reward motivation [16].

Studying the complex behavior of the brain from a brain network perspective will likely improve our understanding of brain function in health and disease states. Since animal models provide an understanding of disease progression, treatment and repair, research on extending brain networks to animal models has attracted increasing attention. Recent studies on brain network changes associated with aging have shown that node betweenness changes significantly with increasing aging [7]. Consistent with our study, the efficiency of information transfer in the left superior colliculus and the right parietal association cortex of aged rats was significantly lower than that of young rats.

The superior colliculus is primarily involved in integrating multisensory information that serves to guide and coordinate orienting motor responses toward visual and auditory signals in space [17]. Typically, visual and auditory impairments are signs of aging, and aging can have a detrimental effect on complex audiovisual interactions. Costa et al. [18] performed extracellular single-unit recording in the superior colliculus of anesthetized Sprague–Dawley adult (10-12 months) and aged (21-22 months) rats. The results showed that the audiovisual interactions in the superior colliculus were more numerous in adult rats (38%) than in aged rats. Moreover, spectral and temporal auditory processing in the superior colliculus is also impaired during aging [19]. In the present study, the significantly lower *BC* in the left superior colliculus of aged rats was thought to be associated with decreased audiovisual interactions with age.

The parietal association cortex, also known as the posterior parietal/anterior medial cortex, is a multichannel sensory region involved in spatial navigation, spatial memory, and associative tasks between different sensory patterns in rats [20, 21]. Meanwhile, the parietal association cortex is part of the retrosplenial cortex, an important cortical region that is closely related to the sensory-cognitive network [22]. As aging is characterized by cognitive impairment, our finding that the metabolic brain network of aged rats had a significantly lower *degree* in the right parietal association cortex would be consistent with its cognitive role. Cognitive impairments, such as learning and memory, are well-known features of the aging process [23, 24]. Rodent models have been frequently used in aging research to study the biochemical and functional effects of aging [25]. Compared with young rats, aged rats show poor spatial learning ability and behavioral flexibility, and the brain volume is correspondingly decreased in cognitive brain areas [26, 27]. Based on brain network analysis, our findings suggest that rats and humans exhibit similar changes in cognitive-related brain regions with age, providing further support for assessing whether changes in topological properties are associated with the behavioral effects of aging.

However, aging in healthy humans typically involves changes in the prefrontal and selective temporal brain regions, according to magnetic resonance imaging (MRI) studies [5, 28]. The characteristics of the aging brain structural network in rats are mainly manifested in the prefrontal/insula and temporal association/perirhinal cortices, as well as the cerebellum and olfactory bulb [2]. It is worth noting that there are some differences between the results of this study and previous studies, which may be due to different neuroimaging methods, nonhuman animal models and analysis methods of network properties [7].

The brain responds robustly to physical damage. Previous studies have indicated that human brain networks are remarkably resilient to different types of lesions compared to other types of complex networks, such as random or scale-free networks [29]. Furthermore, previous findings suggested that network-level robustness might serve as a biomarker of age-related cognitive decline in normal middle-aged individuals decades before the onset of overt cognitive impairment [30]. That said, exploring age-related brain robustness could provide a basis for distinguishing healthy aging from the early stages of degenerative neurological diseases, such as Alzheimer’s disease. In general, the complex network robustness relies heavily on its organizational structure and attack nature [29]. For the latter, two methods are usually applied: random deletion of and targeted attacks on nodes/edges according to their centrality in the network [31]. Damage to specific regions of the brain or their connections is simulated by removing nodes or edges [32]. In addition to the computational lesion study, a further empirical study might also be required. However, it has been difficult to quantify the extent of this virtual damage *in vivo*. Indeed, the node elimination strategy is appropriate for simulating damage to specific brain regions in computational lesion studies [33]. Then, the robustness of the network is usually analyzed based on the ability of the graph to remain in one part despite the removal of elements [29]. Previous studies examining the robustness properties of human networks have shown that the brain networks of the aged group are just as resilient to random failures as those of the young group but more vulnerable to targeted attacks [7]. In this study, there was no significant difference in the size of the largest connected component between aged and young rats under random or targeted attacks. Additionally, we found that the global properties of the remaining networks of aged rats were significantly decreased after random failure (*E_loc_ and Cp*) or targeted attack (*E_loc_*), although there was no significant difference in the global properties of the intact brain networks between the aged rats and young rats. The changes in network parameters can reflect the interruption in the overall performance of the network, such as stability and robustness [7]. In cerebral terms, aging begins long before symptoms manifest. Aging causes gradual degeneration of the myelin sheaths that surround certain nerves, which in turn leads to a decline in the function of neurons and the functional connections between them. Even without symptoms of dysfunction, the network robustness of the normal aging brain has become abnormal. The results of our study were consistent with previous studies on the network robustness of the aging brain; that is, the brain networks of the aged group showed a significant decrease in global efficiency against simulated attacks [34], suggesting that aging brains are vulnerable to severe brain dysfunction even with minor damage. Consistently, human studies have reported that network integrity is decreased in healthy aging, but this decrease is accelerated in Alzheimer's networks, with specific systems hit the hardest [35].

As mentioned above, we investigated differences in the brain metabolic network between aged rats and young rats based on 18F-FDG PET imaging, involving topological properties and network robustness. The results showed decreased regional network measures and vulnerable robustness of brain metabolic networks in aged rats. The findings support the idea that aged rats have similar aging-related changes in the brain metabolic network to the human brain, which can be used as a model for aging studies to provide targets for potential therapies that promote healthy aging.

In addition, understanding the molecular and biological mechanisms of aging will be a key step in preventing, slowing and treating age-related diseases, such as Alzheimer's disease (AD) and Parkinson's disease (PD) [36]. However, there is no gold standard tool for evaluating healthy aging, nor is there a single indicator that can be used as a sensitive and specific biomarker of aging [37]. Currently, epigenetic clocks, a method for using human DNA methylation data to develop biomarkers of aging, have been noted as the most successful aging biomarkers [38]. With the rising prevalence of neurodegenerative diseases of aging, such as AD and PD, several studies have explored epigenetic clocks in brain tissue [39, 40]. This study showed age-related topological changes in some brain regions of the metabolic network, which provided an important basis for further locating epigenetic clocks in brain tissue. Furthermore, these findings, when combined with recently identified molecular and DNA-based markers, have greater potential to improve the prediction of healthy aging and contribute to aging, being better described as a multifactorial interactive process between biological and molecular mechanisms.

There are some limitations to this study. Given the more complex structure and function of the human brain, the study of aged rats might not fully reflect the effects of aging on the human brain. However, it opens the possibility of aging-related research at multiple macro- and microlevels, since the brain of aged rats had similar aging-related changes to the human brain. Another potential limitation was that only female rats were selected as subjects. In the future, we will further explore the sex differences of brain metabolic networks between male and female rats. In addition, it was only an observational study with a cross-sectional comparison. A longitudinal comparison of aging-related brain network changes in rats still needs further exploration.

## MATERIALS AND METHODS

### Rats

In the study, 24 healthy aged female Sprague–Dawley (SD) rats (weight 350–380 g, age 18 months) and 24 healthy young female SD rats (weight 180–200 g, age 8 weeks) were included. Rats were raised under laboratory conditions of 21° C–23° C with a 12-h light-dark cycle and given sufficient food and water for 1 week before the examination.

All rats were obtained from the Shanghai Slack Laboratory Animal Limited Liability Company (Shanghai, China). All procedures for animals were carried out in accordance with the Guide for the Care and Use of Laboratory Animals (US National Institutes of Health) and approved by the Animal Ethical Committee of Shanghai University of Traditional Chinese Medicine.

### 18 F-FDG PET/CT scan

Scanning was conducted on a PET/CTR4 bed (Siemens Inc., USA). To enhance brain absorption of the tracer, all rats were fasted overnight before scanning. After 0.5 mCi 18F-FDG was injected through the tail vein, the rats were placed in a quiet space for an uptake period of 40 minutes. During scanning, halothane gas was used to anesthetize the rats, with a 5% induction and a 1.5% maintenance dose. After acquisition, the attenuation correction was automatically carried out, and images were obtained in a 128 × 128 matrix and recombined in OSEM3D mode. PET/CT acquisition showed the following parameters: spherical tube voltage (80 kV), current (500 μA), and time (492 s).

All rats were in a normal active state before 18F-FDG PET/CT scanning. One aged rat was excluded from the final analysis because of poor image quality. Thus, 23 aged rats and 24 young rats were included in the final data analysis.

### Data preprocessing

Data preprocessing was performed using the Statistical Parametric Mapping 8 toolbox (SPM 8; http://www.fil.ion.ucl.ac.uk/spm/) based on the MATLAB 2013b platform (Mathworks, Inc., Natick, MA, USA). Preprocessing was performed as previously reported [41]. The DICOM-format PET/CT images were adapted into the NIFTI format by ImageJ software (Image Processing and Analysis in Java, National Institutes of Health, Bethesda, MD, USA). A hand drawing mask was applied to extract the rats’ PET images of the skull-stripped brain. The orientation of these images was modified by resetting the origin and adjusting pitch/roll/yaw parameters according to a standard template. To fit the algorithm in SPM8, the voxels were upscaled by a factor of 10. Accordingly, each brain PET image was normalized and resampled to a resolution of 2.06 × 2.06 × 2 mm^3^. In addition, the images were smoothed by a full width at half maximum (FWHM) twice the voxel size (FWHM = 4 mm). Finally, the 18F-FDG uptake value of each voxel was globally normalized by the mean uptake value of the whole brain [42].

### Construction and property analysis of the metabolic brain network

**Figure 9:**
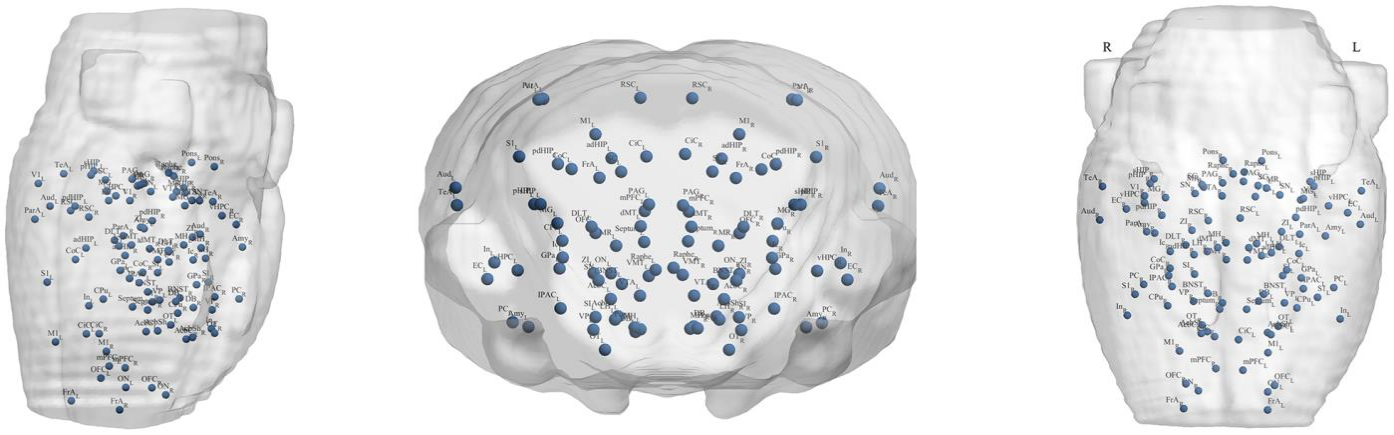
Locations of the 96 brain regions.

**Figure 10:**
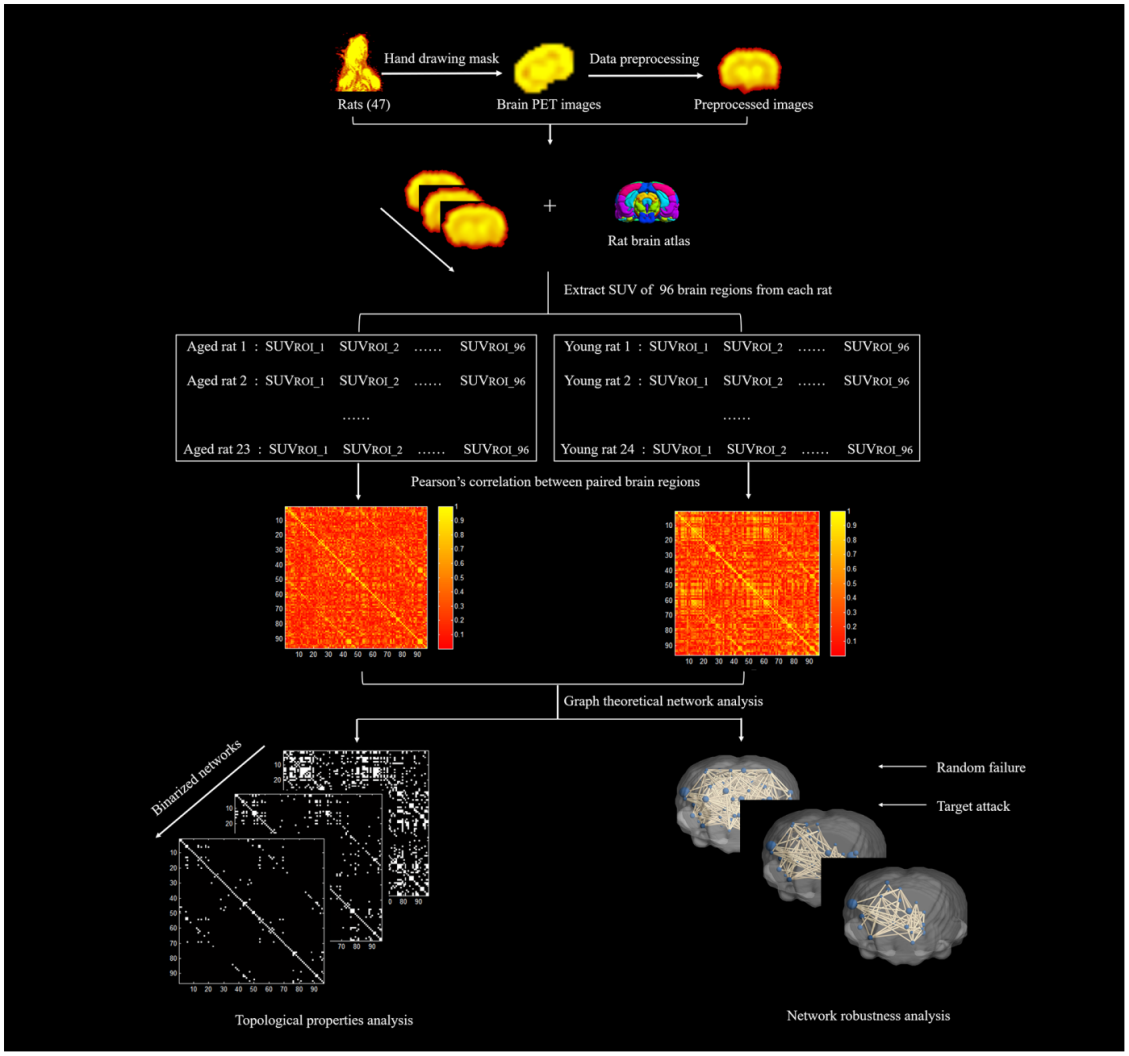
The diagram of network construction and analysis.

Metabolic brain network construction and network property analysis were processed with the Brain Connectivity Toolbox (BCT, version 2017-15-01, http://www.brain-connectivity-toolbox.net/). Based on the standard rat brain template including 96 brain regions [43], the standard uptake value (SUV) of each brain region of all rats was extracted, and the Pearson correlation coefficient of each two brain regions was calculated to generate the group-level metabolic brain network in an intersubject manner. The nodes of the network were defined as the brain regions. The edges of the network were considered to be the correlation between each pair of brain regions. Figure 9 shows the locations of the 96 brain regions, and the explanation of the abbreviated region names is given in Table 1. To ensure that all of the networks had the same number of edges, the specific range of sparsity (0.05-0.5) at an interval of 0.01 was set for the correlation matrix [44], resulting in a set of undirected and unweighted binary networks. Finally, we explored the differences in topological properties of the metabolic brain network in aged rats and young rats by using graph theory network analysis. In addition, a simple analysis of network robustness against random failure and targeted attacks was performed. The diagram of network construction and analysis is shown in Figure 10.

### Global network properties

The topological properties of the global network, including path length (*Lp*), clustering coefficient (*Cp*), global efficiency (*E_glob_*), local efficiency (*E_loc_*) and small-worldness indices (*σ*, *γ*, *λ*), were calculated to describe the network’s global and local information transmission capability [45]. The *Lp* of the network refers to the average of the shortest path’s edges in the network’s node pairs [46]. The *Cp* of a node is the ratio of existing edges to all the others in its direct neighbors. The *Cp* of a network is the mean of *Cp* over all nodes, reflecting the local interconnectivity of the network [46]. *E_glob_* refers to the inverse of all the network nodes’ average shortest *Lp* [45], and *E_loc_* represents that of a node’s nearest neighbors [47]. By comparing the *Cp* and *Lp* to the mean *Cp_rand_* and *Lp_rand_* of 5000 random networks, the normalized *Cp* (*γ*) and normalized *Lp* (*λ*) were calculated. A network’s small-worldness is defined as *σ* = *γ*/*λ*, representing the balance between all of the network nodes’ segregation and integration [48, 49]. When the ratio is greater than 1, the network is considered a small-worldness network.

### Regional network properties

Three topological characteristics of the regional network were used to demonstrate the nodal characteristics: degree (*D*), betweenness centrality (*BC*) and node efficiency (*E_nod_*). *D* refers to the number of neighbors of a given node, reflecting the importance of the node [50]. *BC* is defined as the fraction of shortest paths of a network through a given node, which is an indicator of the importance of a node in the network [51]. *E_nod_* is the average of the inverse of the shortest path between a given node and all other nodes and is an evaluation of regional connectivity [52].

### Network robustness

Network robustness against simulated damage was analyzed by the iterative ‘random’ and ‘targeted’ removal of nodes [7, 53]. A random node attack refers to the continuous removal of a certain proportion of random nodes from the 96 × 96 connectivity matrix. Each random removal of nodes was repeated 40 times. In procedures requiring the targeted node attack, the 96 nodes are arranged in order of *BC* value from high to low. Nodes were removed in order of *BC* value, starting with the nodes of higher connectivity. In the case that information transfer follows the shortest path, *BC* reflects the centrality of nodes in the network. Therefore, the nodes with high centrality have a greater impact on the information transfer in the network. After each removal of nodes, the above global topological measures, including *Lp*, *Cp*, *E_glob_*, *E_loc_*, *σ*, *γ*, and *λ*, were calculated for the resulting networks thresholded at minimum density with full connectivity. In addition, the size of the largest connected component was calculated when nodes were continuously removed. Additionally, the network properties of the resulting networks after each random attack were averaged across 40 repetitions of random removal.

### Statistical analysis

A 5000-repetition nonparametric permutation test was used to determine the between-group differences’ statistical significance. *Metabolic Connectivity Analysis.* We performed statistical comparison on the edges of the metabolic network between groups by 5000 permutation tests. In each repetition, the SUV of the ROIs of all rats was randomly assigned to two new groups with the sample sizes as the original groups, and then the Pearson correlation coefficient of each two brain regions was calculated to generate a new group-level metabolic brain network. After 5000 permutations, we obtained 5000 random intergroup differences in metabolic connectivity. Thus, the *p* value was obtained according to the percentile position of the real intergroup difference in the corresponding permutation distribution. *Graph Theoretical Network Analysis.* First, during each repetition, the SUV values of each rat were randomly assigned to either the aged group or the young group to form two randomized groups with the same number of rats as the original group. Second, the undirected and unweighted binarized network was established for each randomized group based on the new dataset. Third, the network properties of randomized groups were calculated through all sparsity thresholds, and the area under the curve (AUC) corresponding to the sparsity ranges of both groups was calculated in each permutation cycle. Last, the AUC difference between the two groups obtained under 5000 permutations constituted the permutation distribution under the original hypothesis. The *p value* was obtained from the AUC’s actual intergroup difference at the distribution. The above procedure was also used to investigate significant differences in the network robustness between the two groups. After each removal of nodes, the network properties of the two remaining networks were compared by a nonparametric permutation test with 5000 repetitions mentioned above. The significance level for the global network analysis was set at *p* < 0.05. *p* <0.05 after FDR correction for multiple comparisons was the significance level for regional network analysis.

## Supplementary Material

Supplementary File

Supplementary Tables

## References

[1] Damoiseaux JS. Effects of aging on functional and structural brain connectivity. Neuroimage. 2017; 160:32–40. 10.1016/j.neuroimage.2017.01.077 28159687

[2] Alexander GE, Lin L, Yoshimaru ES, Bharadwaj PK, Bergfield KL, Hoang LT, Chawla MK, Chen K, Moeller JR, Barnes CA, Trouard TP. Age-Related Regional Network Covariance of Magnetic Resonance Imaging Gray Matter in the Rat. Front Aging Neurosci. 2020; 12:267. 10.3389/fnagi.2020.00267 33005147 PMC7479213

[3] Bonte S, Vandemaele P, Verleden S, Audenaert K, Deblaere K, Goethals I, Van Holen R. Healthy brain ageing assessed with 18F-FDG PET and age-dependent recovery factors after partial volume effect correction. Eur J Nucl Med Mol Imaging. 2017; 44:838–49. 10.1007/s00259-016-3569-0 27878594

[4] Huang CC, Hsieh WJ, Lee PL, Peng LN, Liu LK, Lee WJ, Huang JK, Chen LK, Lin CP. Age-related changes in resting-state networks of a large sample size of healthy elderly. CNS Neurosci Ther. 2015; 21:817–25. 10.1111/cns.12396 25864728 PMC6493082

[5] Zhao T, Cao M, Niu H, Zuo XN, Evans A, He Y, Dong Q, Shu N. Age-related changes in the topological organization of the white matter structural connectome across the human lifespan. Hum Brain Mapp. 2015; 36:3777–92. 10.1002/hbm.22877 26173024 PMC6869038

[6] Geerligs L, Renken RJ, Saliasi E, Maurits NM, Lorist MM. A Brain-Wide Study of Age-Related Changes in Functional Connectivity. Cereb Cortex. 2015; 25:1987–99. 10.1093/cercor/bhu012 24532319

[7] Liu Z, Ke L, Liu H, Huang W, Hu Z. Changes in topological organization of functional PET brain network with normal aging. PLoS One. 2014; 9:e88690. 10.1371/journal.pone.0088690 24586370 PMC3930631

[8] Rocher AB, Chapon F, Blaizot X, Baron JC, Chavoix C. Resting-state brain glucose utilization as measured by PET is directly related to regional synaptophysin levels: a study in baboons. Neuroimage. 2003; 20:1894–8. 10.1016/j.neuroimage.2003.07.002 14642499

[9] Kalpouzos G, Chételat G, Baron JC, Landeau B, Mevel K, Godeau C, Barré L, Constans JM, Viader F, Eustache F, Desgranges B. Voxel-based mapping of brain gray matter volume and glucose metabolism profiles in normal aging. Neurobiol Aging. 2009; 30:112–24. 10.1016/j.neurobiolaging.2007.05.019 17630048

[10] Pardo JV, Lee JT, Sheikh SA, Surerus-Johnson C, Shah H, Munch KR, Carlis JV, Lewis SM, Kuskowski MA, Dysken MW. Where the brain grows old: decline in anterior cingulate and medial prefrontal function with normal aging. Neuroimage. 2007; 35:1231–7. 10.1016/j.neuroimage.2006.12.044 17321756 PMC1913629

[11] de Villers-Sidani E, Merzenich MM. Lifelong plasticity in the rat auditory cortex: basic mechanisms and role of sensory experience. Prog Brain Res. 2011; 191:119–31. 10.1016/B978-0-444-53752-2.00009-6 21741548

[12] Mohamed ME, El-Shaarawy EA, Youakim MF, Shuaib DM, Ahmed MM. Aging changes in the retina of male albino rat: a histological, ultrastructural and immunohistochemical study. Folia Morphol (Warsz). 2019; 78:237–58. 10.5603/FM.a2018.0075 30155876

[13] Lessard-Beaudoin M, Yu-Taeger L, Laroche M, Singer E, Riess O, Nguyen HH, Graham RK. Olfactory bulb atrophy and caspase activation observed in the BACHD rat models of Huntington disease. Neurobiol Dis. 2019; 125:219–31. 10.1016/j.nbd.2019.02.002 30738141

[14] Tsopelas C, Stewart R, Savva GM, Brayne C, Ince P, Thomas A, Matthews FE, and Medical Research Council Cognitive Function and Ageing Study. Neuropathological correlates of late-life depression in older people. Br J Psychiatry. 2011; 198:109–14. 10.1192/bjp.bp.110.078816 21282780

[15] Proaño SB, Meitzen J. Estradiol decreases medium spiny neuron excitability in female rat nucleus accumbens core. J Neurophysiol. 2020; 123:2465–75. 10.1152/jn.00210.2020 32432511 PMC7311729

[16] Bowen HJ, Gallant SN, Moon DH. Influence of Reward Motivation on Directed Forgetting in Younger and Older Adults. Front Psychol. 2020; 11:1764. 10.3389/fpsyg.2020.01764 32849044 PMC7411084

[17] Lau C, Manno FA, Dong CM, Chan KC, Wu EX. Auditory-visual convergence at the superior colliculus in rat using functional MRI. Annu Int Conf IEEE Eng Med Biol Soc. 2018; 2018:5531–6. 10.1109/EMBC.2018.8513633 30441590

[18] Costa M, Piché M, Lepore F, Guillemot JP. Age-related audiovisual interactions in the superior colliculus of the rat. Neuroscience. 2016; 320:19–29. 10.1016/j.neuroscience.2016.01.058 26844390

[19] Costa M, Lepore F, Guillemot JP. Spectral and temporal auditory processing in the superior colliculus of aged rats. Neurobiol Aging. 2017; 57:64–74. 10.1016/j.neurobiolaging.2017.05.015 28605641

[20] Mesulam MM. From sensation to cognition. Brain. 1998; 121:1013–52. 10.1093/brain/121.6.1013 9648540

[21] Torrealba F, Valdés JL. The parietal association cortex of the rat. Biol Res. 2008; 41:369–77. 10.4067/S0716-97602008000400002 19621117

[22] Wang J, Nie B, Duan S, Zhu H, Liu H, Shan B. Functionally Brain Network Connected to the Retrosplenial Cortex of Rats Revealed by 7T fMRI. PLoS One. 2016; 11:e0146535. 10.1371/journal.pone.0146535 26745803 PMC4706345

[23] Cohen RA, Marsiske MM, Smith GE. Neuropsychology of aging. Handb Clin Neurol. 2019; 167:149–80. 10.1016/B978-0-12-804766-8.00010-8 31753131

[24] Park DC, Reuter-Lorenz P. The adaptive brain: aging and neurocognitive scaffolding. Annu Rev Psychol. 2009; 60:173–96. 10.1146/annurev.psych.59.103006.093656 19035823 PMC3359129

[25] Hoekzema E, Herance R, Rojas S, Pareto D, Abad S, Jiménez X, Figueiras FP, Popota F, Ruiz A, Torrent È, Fernández-Soriano FJ, Rocha M, Rovira M, et al. The effects of aging on dopaminergic neurotransmission: a microPET study of [11C]-raclopride binding in the aged rodent brain. Neuroscience. 2010; 171:1283–6. 10.1016/j.neuroscience.2010.10.012 20937365

[26] Hamezah HS, Durani LW, Ibrahim NF, Yanagisawa D, Kato T, Shiino A, Tanaka S, Damanhuri HA, Ngah WZ, Tooyama I. Volumetric changes in the aging rat brain and its impact on cognitive and locomotor functions. Exp Gerontol. 2017; 99:69–79. 10.1016/j.exger.2017.09.008 28918364

[27] Mota C, Taipa R, das Neves SP, Monteiro-Martins S, Monteiro S, Palha JA, Sousa N, Sousa JC, Cerqueira JJ. Structural and molecular correlates of cognitive aging in the rat. Sci Rep. 2019; 9:2005. 10.1038/s41598-019-39645-w 30765864 PMC6376121

[28] Colangeli S, Boccia M, Verde P, Guariglia P, Bianchini F, Piccardi L. Cognitive Reserve in Healthy Aging and Alzheimer’s Disease: A Meta-Analysis of fMRI Studies. Am J Alzheimers Dis Other Demen. 2016; 31:443–9. 10.1177/1533317516653826 27307143 PMC10852844

[29] Aerts H, Fias W, Caeyenberghs K, Marinazzo D. Brain networks under attack: robustness properties and the impact of lesions. Brain. 2016; 139:3063–83. 10.1093/brain/aww194 27497487

[30] Korthauer LE, Zhan L, Ajilore O, Leow A, Driscoll I. Disrupted topology of the resting state structural connectome in middle-aged APOE ε4 carriers. Neuroimage. 2018; 178:295–305. 10.1016/j.neuroimage.2018.05.052 29803958 PMC6249680

[31] Bullmore E, Sporns O. Complex brain networks: graph theoretical analysis of structural and functional systems. Nat Rev Neurosci. 2009; 10:186–98. 10.1038/nrn2575 19190637

[32] Albert R, Barabasi AL. Statistical mechanics of complex networks. Rev Mod Phys. 2002; 74:47–97. 10.1103/RevModPhys.74.47

[33] Shu P, Zhu H, Jin W, Zhou J, Tong S, Sun J. The Resilience and Vulnerability of Human Brain Networks Across the Lifespan. IEEE Trans Neural Syst Rehabil Eng. 2021; 29:1756–65. 10.1109/TNSRE.2021.3105991 34410925

[34] Gomez-Ramirez J, Li Y, Wu Q, Wu J. A Quantitative Study of Network Robustness in Resting-State fMRI in Young and Elder Adults. Front Aging Neurosci. 2016; 7:256. 10.3389/fnagi.2015.00256 26869917 PMC4737864

[35] Dennis EL, Thompson PM. Functional brain connectivity using fMRI in aging and Alzheimer’s disease. Neuropsychol Rev. 2014; 24:49–62. 10.1007/s11065-014-9249-6 24562737 PMC4109887

[36] Shireby GL, Davies JP, Francis PT, Burrage J, Walker EM, Neilson GW, Dahir A, Thomas AJ, Love S, Smith RG, Lunnon K, Kumari M, Schalkwyk LC, et al. Recalibrating the epigenetic clock: implications for assessing biological age in the human cortex. Brain. 2020; 143:3763–75. 10.1093/brain/awaa334 33300551 PMC7805794

[37] Wagner KH, Cameron-Smith D, Wessner B, Franzke B. Biomarkers of Aging: From Function to Molecular Biology. Nutrients. 2016; 8:338. 10.3390/nu8060338 27271660 PMC4924179

[38] Jylhävä J, Pedersen NL, Hägg S. Biological Age Predictors. EBioMedicine. 2017; 21:29–36. 10.1016/j.ebiom.2017.03.046 28396265 PMC5514388

[39] Lu AT, Hannon E, Levine ME, Crimmins EM, Lunnon K, Mill J, Geschwind DH, Horvath S. Genetic architecture of epigenetic and neuronal ageing rates in human brain regions. Nat Commun. 2017; 8:15353. 10.1038/ncomms15353 28516910 PMC5454371

[40] Grodstein F, Lemos B, Yu L, Iatrou A, De Jager PL, Bennett DA. Characteristics of Epigenetic Clocks Across Blood and Brain Tissue in Older Women and Men. Front Neurosci. 2021; 14:555307. 10.3389/fnins.2020.555307 33488342 PMC7817909

[41] Huo BB, Shen J, Hua XY, Zheng MX, Lu YC, Wu JJ, Shan CL, Xu JG. Alteration of metabolic connectivity in a rat model of deafferentation pain: a 18F-FDG PET/CT study. J Neurosurg. 2019; 132:1295–303. 10.3171/2018.11.JNS181815 30835695

[42] Hou AL, Zheng MX, Hua XY, Huo BB, Shen J, Xu JG. Electroacupuncture-Related Metabolic Brain Connectivity in Neuropathic Pain due to Brachial Plexus Avulsion Injury in Rats. Front Neural Circuits. 2020; 14:35. 10.3389/fncir.2020.00035 32625066 PMC7313422

[43] Schwarz AJ, Danckaert A, Reese T, Gozzi A, Paxinos G, Watson C, Merlo-Pich EV, Bifone A. A stereotaxic MRI template set for the rat brain with tissue class distribution maps and co-registered anatomical atlas: application to pharmacological MRI. Neuroimage. 2006; 32:538–50. 10.1016/j.neuroimage.2006.04.214 16784876

[44] Bullmore ET, Bassett DS. Brain graphs: graphical models of the human brain connectome. Annu Rev Clin Psychol. 2011; 7:113–40. 10.1146/annurev-clinpsy-040510-143934 21128784

[45] Latora V, Marchiori M. Efficient behavior of small-world networks. Phys Rev Lett. 2001; 87:198701. 10.1103/PhysRevLett.87.198701 11690461

[46] Watts DJ, Strogatz SH. Collective dynamics of ‘small-world’ networks. Nature. 1998; 393:440–2. 10.1038/30918 9623998

[47] Shah C, Liu J, Lv P, Sun H, Xiao Y, Liu J, Zhao Y, Zhang W, Yao L, Gong Q, Lui S. Age Related Changes in Topological Properties of Brain Functional Network and Structural Connectivity. Front Neurosci. 2018; 12:318. 10.3389/fnins.2018.00318 29867329 PMC5962656

[48] Humphries MD, Gurney K. Network ‘small-world-ness’: a quantitative method for determining canonical network equivalence. PLoS One. 2008; 3:e0002051. 10.1371/journal.pone.0002051 18446219 PMC2323569

[49] Li Y, Wang Y, Wang Y, Wang H, Li D, Chen Q, Huang W. Impaired Topological Properties of Gray Matter Structural Covariance Network in Epilepsy Children With Generalized Tonic-Clonic Seizures: A Graph Theoretical Analysis. Front Neurol. 2020; 11:253. 10.3389/fneur.2020.00253 32373045 PMC7176815

[50] Xin Z, Chen X, Zhang Q, Wang J, Xi Y, Liu J, Li B, Dong X, Lin Y, Zhang W, Chen J, Luo W. Alteration in topological properties of brain functional network after 2-year high altitude exposure: A panel study. Brain Behav. 2020; 10:e01656. 10.1002/brb3.1656 32909397 PMC7559604

[51] Rubinov M, Sporns O. Complex network measures of brain connectivity: uses and interpretations. Neuroimage. 2010; 52:1059–69. 10.1016/j.neuroimage.2009.10.003 19819337

[52] Jiang W, Zhao Z, Wu Q, Wang L, Zhou L, Li D, He L, Tan Y. Study on brain structure network of patients with delayed encephalopathy after carbon monoxide poisoning: based on diffusion tensor imaging. Radiol Med. 2021; 126:133–41. 10.1007/s11547-020-01222-x 32557108

[53] Mohan A, De Ridder D, Vanneste S. Robustness and dynamicity of functional networks in phantom sound. Neuroimage. 2017; 146:171–87. 10.1016/j.neuroimage.2016.04.033 27103139

