# Supplementary material for: Age-related alterations of brain metabolic network based on [18F]FDG-PET of rats
Source: Aging (Albany NY). 2022 Jan 25;14(2):923–42. doi: 10.18632/aging.203851 (PMC8833125; doi:10.18632/aging.203851)
Supplement: Supplementary File [file aging-14-203851-s001.pdf]

SUPPLEMENTARY FILE

MATERIALS AND METHODS

Leave-one-out (LOO)

First of all, one group-level metabolic network was constructed based on [18F]Fluoro-deoxyglucose with positron emission tomography (18F-FDG PET) data of a group of subjects [1, 2]. Then, data from one subject was removed and the group-level metabolic network was constructed based on the remaining dataset. Finally, the Mantel test was performed to detect the similarity between the group-level network after removing any one of the subjects and the original group-level network. A higher similarity was considered as a lower variability.

The Mantel test was proposed in 1967 to test the correlation between two matrices. The calculation formula is:

$$MantelTest(P,M) = \frac{1}{n-1}\sum_{i=1}^{n}\sum_{j=1}^{n}\frac{p_{ij}-\overline{p}}{S_p}\bullet\frac{m_{ij}-\overline{m}}{S_m}$$

In this study, n was the number of subjects, M represented the original group-level brain metabolic network matrix. After removing any one of the subjects, the group-level brain metabolic network based on the remaining dataset (P) was constructed. S_p and S_m were the standard variances of P and M respectively. The coefficient of Mantel test (r) ranged from -1 to 1. The closer the absolute value of the coefficient was to 1, the more similar M and P were. Furthermore, a 1000-time permutation test was used to determine the coefficient's statistical significance.

RESULTS

The Mantel Test showed significantly positive correlation between the original group-level brain metabolic network matrix and the network matrix constructed after removing any one of the subjects in both the aged and young groups (all $p < 0.001$). (Supplementary Tables 1, 2).

DISCUSSION

This study also investigated inter-individual variability among the used subjects. There was a high correlation between the original group-level brain metabolic network matrix and the network matrix constructed after removing any one of the subjects in both the aged and young groups. In other words, inter-individual variability among the used subjects had less impact on the results. We believed that the results were closely related to the high homogeneity of the rat models which could provide tools to examine the effect of singular pathologies, and the interaction of multiple factors in a well-controlled environment.

REFERENCES

1. Liu Z, Ke L, Liu H, Huang W, Hu Z. Changes in topological organization of functional PET brain network with normal aging. PLoS One. 2014; 9:e88690. https://doi.org/10.1371/journal.pone.0088690 PMID:24586370

2. Huang SY, Hsu JL, Lin KJ, Liu HL, Wey SP, Hsiao IT; Alzheimer's Disease Neuroimaging Initiative. Characteristic patterns of inter- and intra-hemispheric metabolic connectivity in patients with stable and progressive mild cognitive impairment and Alzheimer's disease. Sci Rep. 2018; 8:13807. https://doi.org/10.1038/s41598-018-31794-8 PMID:30218083
