# Supplementary material for: Age-related alterations of brain metabolic network based on [18F]FDG-PET of rats
Source: Aging (Albany NY). 2022 Jan 25;14(2):923–42. doi: 10.18632/aging.203851 (PMC8833125; doi:10.18632/aging.203851)
Supplement: Supplementary Tables [file aging-14-203851-s002.pdf]

SUPPLEMENTARY TABLES

Supplementary Table 1. Results of inter-individual variability among the aged rats by the leave-one-out(LOO) method.

No.	r	p
1	0.998	<0.001
2	0.998	<0.001
3	0.997	<0.001
4	0.997	<0.001
5	0.990	<0.001
6	0.971	<0.001
7	0.991	<0.001
8	0.999	<0.001
9	0.997	<0.001
10	0.998	<0.001
11	0.987	<0.001
12	0.995	<0.001
13	0.987	<0.001
14	0.989	<0.001
15	0.988	<0.001
16	0.988	<0.001
17	0.950	<0.001
18	0.992	<0.001
19	0.998	<0.001
20	0.996	<0.001
21	0.998	<0.001
22	0.979	<0.001
23	0.997	<0.001

No., the number of the removing rat during the leave-one-out procedure; r, the correlation coefficient of the Mantel test between the group-level metabolic network after removing the specific rat and the original network; p, p value of the Mantel's r statistics by 1000-time permutational tests ($p<0.05$ is considered as significant).

Supplementary Table 2. Results of inter-individual variability among the young rats by the leave-one-out (LOO) method.

No.	r	p
1	0.998	<0.001
2	0.993	<0.001
3	0.995	<0.001
4	0.998	<0.001
5	0.992	<0.001
6	0.996	<0.001
7	0.986	<0.001
8	0.998	<0.001
9	0.999	<0.001
10	0.994	<0.001
11	0.998	<0.001
12	0.997	<0.001
13	0.998	<0.001
14	0.994	<0.001
15	0.995	<0.001
16	0.974	<0.001
17	0.994	<0.001
18	0.996	<0.001
19	0.996	<0.001
20	0.983	<0.001
21	0.989	<0.001
22	0.993	<0.001
23	0.992	<0.001
24	0.992	<0.001

No., the number of the removing rat during the leave-one-out procedure; r, the correlation coefficient of the Mantel test between the group-level metabolic network after removing the specific rat and the original network; p, p value of the Mantel's r statistics by 1000-time permutational tests ($p<0.05$ is considered as significant).
